# Editorial

**Published:** 1914-05

**Authors:** 


### EDITORIAL

The Business Office of the *Journal-Record* is 23 West Alabama Street.

The Editorial Office is 1014-15 Atlanta National Bank Building.

Address all Business Communications to *Journal-Record of Medicine*, 23 West Alabama Street.

Make remittances payable to *The Journal-Record of Medicine*.

On matters pertaining to the Editorial and Original Communications, address Edgar G. Ballenger, M. D., Atlanta, Ga.

Reprints of Original Articles will be furnished at cost price. Requests for the same should always be made in THE MANUSCRIPT.

We will present, postpaid, on request to each contributor of an original article, twenty (20) marked copies of *The Journal-Record of Medicine* containing such article.

### TYPHUS FEVER—THE PLOTZ BACILLUS

Early in the month, the New York Times published a statement that Dr. Harry Plotz, a young interne of Mt. Sinai Hospital in New York, had been working in the laboratory and studying the relations between Brills Disease and Typhus Fever until he had determined that they were the same thing and caused by a germ which he has isolated and proved. The article also stated that, the preliminary paper describing the experimental work would be read at the meeting of the American Association of Physicians assembled at Atlantic City on the 13th. Later on the same paper stated that Dr. Plotz was not allowed to read the paper because it had been previously announced in the public press. A further statement made it clear that Plotz was not party to the publication. An editorial called the proceedings "Mediaeval Ethics" and spoke of "narrow-minded persons," who seemed to be acting to deprive Plotz of the credit due him. The Times declared its independence of the professional ethics involved and said it would continue to print such medical matters as appealed to it as news. Still later came the apology that Plotz was not prevented from reading his paper by any other circustances that that it was not reached in the program and that several other eminent men were "silenced" in the same way.

Then came expressions of annoyance from some of the professional journals that there had been lay comment upon such an important matter prior to having it passed upon by expert critics.

Altogether Dr. Plotz has had no little advertising even if it may have been coupled with some mental irritation. He certainly is entitled to all the recognition and praise that can be given to him and these should come unstinted by any jealousy, particularly of code rules.

Colonel Gorgas has shown that Typhus fever is communicated by the bites of lice and he has done a great deal to secure freedom from epidemics of the fever by restricting lice-bearing animals. His sanitary measures have been far reaching and successful, but all the time he has been handicapped by a lack of fundamental knowledge of the disease itself. Dr. Plotz now comes forward with his preliminary report, carefully describing the bacillus so that all investigators may go over the ground, and promising a further report in the near future. He has made his studies from cases gathered at the port of New York where there was no doubt as to the diagnosis. He has confirmed and checked his work by the observation of other men in the laboratory to whom he gives credit.

He has made what we feel safe in recognizing as a great discovery in the prevention of disease and one that should place him in the front rank of investigators of the world. His hope to offer us a preventive of the disease whether as a serum or antitoxin is reasonable and we share it with him.

Who are the most concerned as to the control of this devasting disease, the doctors or the patients? One would hardly care to answer the question put in that form because the interests can not be differentiated. It is our observation that the popularizing of information as to the prevention of disease is necessary before the people will be governed by it to any valuable extent, and that health officers wherever they work are tell-everyone who will listen what is best for the communities in which they live. There as been so much information of this character given out through circulars and lectures that the interest the people at large have in them is an intelligent and thoughtful one. The people want to know and they are constantly asking their doctors for information as to the etiology and prevention of the great, well-known diseases. Since the national guard has been vaccinated against typhoid, there have been numerous requests by well informed men for the same protection to their families. The men of the world, the laity, have taken thought in the matter. They are entitled to know, and no shroud of mysticism should prevent their clarity of vision.

On matters of great public moment we can see no reason why the public press should not have abundant opportunity to print the latest discoveries and to lead the people into discussion of them. If the daily papers score a "beat" over the weekly or monthly medical journals, no one cares five minutes about it unless someone loses his saving grace of humor and stands on a high pedestal to quarrel about it. The medical journal will be along in a few days anyhow and if it contains information that will permit its readers to give correct information and clear explanations to the men who are questioning their family doctors as to the latest discovery, the medical paper will have served its purpose and sometimes filled a long-felt want.

Once more we congratulate Dr. Plotz upon his work. We believe that to him and to all other young men who are making careful studies of the mysteries of life there should come prompt and free recognition of their endeavors and all the encouragement of fame should be their reward. R. R. D.

